# Supplementary material for: LIPG-mediated regulation of lipid deposition and proliferation in goat intramuscular preadipocytes involves the PPARα signaling pathway
Source: PLoS One. 2025 Feb 13;20(2):e0317953. doi: 10.1371/journal.pone.0317953 (PMC11825097; doi:10.1371/journal.pone.0317953)
Supplement: S1 Table — (PDF) [file pone.0317953.s001.pdf]

S1 Table Summary of genes, peimers and product sizes for RT-qPCR

Gene	Description	GenBank ID number	Primer name	Primer sequence(5'to3')	Size（bp）	application
LIPG	Endothelial Lipase	xM_005697191.3	Sense	CTTGGACCGCTGGAAAC	2174	cloning
			Antisense	GGGAGCAGGACGGAATA		
			Sense	ATTTAACCTCCGCACCTCG	139	RT-qPCR
			Antisense	GCCACTCATCGTCCATCC		
**Fatty acid activation and elongation**						
ACSL1	Acyl-CoA Synthetase Long Chain Family Member 1	XM_005698718	Sense	TGACTGTTGCTGGAGACTGG	220	RT-qPCR
			Antisense	CAGCCGTCTTTATCCAGAGC		
ACSS2	Acyl-CoA Synthetase Short Chain Family Member 2	XM_018057751	Sense	GGCGAATGCCTCTACTGCTT	100	RT-qPCR
			Antisense	GGCCAATCTTTTCTCTAATCTGCTT		
ACC	Acetyl-CoA Carboxylase Alpha	NM_174224.2	Sense	CTCCAACCTCAACCACTACGG	171	RT-qPCR
			Antisense	GGGGAATCACAGAAGCAGCC		
FABP3	Fatty Acid Binding Protein 3	NM_001285701	Sense	GATGAGACCACGGCAGATG	120	RT-qPCR
			Antisense	GTCAACTATTTCCCGCACAAG		
CD36	scavenger receptor B2	X91503	Sense	GTACAGATGCAGCCTCATTTCC	81	RT-qPCR
			Antisense	TGGACCTGCAAATATCAGAGGA		
**Fatty acid desaturation and elongation**						
FADS1	Fatty Acid Desaturase 1	XM_018043052.1	Sense	GGTGGACTTGGCCTGGATG	101	RT-qPCR
			Antisense	TGACCATGAAGACAAGCCCC		
ELOVL6	Long-chain fatty acid family member 6	NM_001314257.1	Sense	GGAAGCCTTTAGTGCTCTGGTC	205	RT-qPCR
			Antisense	ATTGTATCTCCTAGTTCGGGTGC		
**TAG synthesis**						
AGPAT6		JI861797.1	Sense	AAGCAAGTTGCCCATCCTCA	101	RT-qPCR

	1-acylglycerol-3-phosphate O-acyltransferase 6		Antisense	AAACTGTGGCTCCAATTTCGA		
DGAT1	Diacylglycerol O-Acyltransferase 1	NM_174693	Sense<br>Antisense	CACTGGGACCTGAGGTGTC<br>GCATCACCACACACCAATTCA	111	RT-qPCR
FASN	Fatty Acid Synthase	DQ915966.3	Sense<br>Antisense	GGGCTCCACCACCGTGTTCCA<br>GCTCTGCTGGGCCTGCAGCTG	226	RT-qPCR
**TAG lipolasis**						
LPL	Lipoprotein Lipase	DQ997818	Sense<br>Antisense	AGGACACTTGCCACCTCATTC<br>TTGGAGTCTGGTTCCCTCTTGTA	169	RT-qPCR
ATGL	Phospholipase Domain Containing 2	GQ918145	Sense<br>Antisense	GGAGCTTATCCAGGCCAATG<br>TGCGGGCAGATGTCACTCT	180	RT-qPCR
HSL	Hormone-sensitive lipase	EU273879	Sense<br>Antisense	GGGAGCACTACAAACGCAACG<br>TGAATGATCCGCTCAAACTCG	118	RT-qPCR
**Fatty acid oxidation**						
ACOX1	Acyl-CoA Oxidase 1	NM_00103528	Sense<br>Antisense	CGAGTTCATTCTCAACAGTCCT<br>GCATCTTCAAGTAGCCATTATCC	211	RT-qPCR
CPT1A	Carnitine Palmitoyltransferase 1A	XM_018043311.1	Sense<br>Antisense	TGACGGCTCTGGCACAAGAT<br>CGCGAAGTAGTTGCTATTCAC	164	RT-qPCR
CPT1B	Carnitine Palmitoyltransferase 1B	NM_001009259	Sense<br>Antisense	ACGAGGAGTCTCACCACTACG<br>GTGTGAAGGACTTGTCGAACCA	111	RT-qPCR
**Fatty acid regulators**						
PPARα	Peroxisome proliferator-activated receptor-α	HM600811.1		TACTCTCGGCAGACTTCCTAC<br>CCTCCTCACATCTGTCATACAC	201	RT-qPCR
**Internal reference**						
UXT	Ubiquitously expressed prefoldin like chaperone	XP_005700899.1	Sense<br>Antisense	GCAAGTGGATTTGGGCTGTAAC<br>ATGGAGTCCTTGGTGAGGTTGT	125	RT-qPCR
