# Supplementary material for: LIPG-mediated regulation of lipid deposition and proliferation in goat intramuscular preadipocytes involves the PPARα signaling pathway
Source: PLoS One. 2025 Feb 13;20(2):e0317953. doi: 10.1371/journal.pone.0317953 (PMC11825097; doi:10.1371/journal.pone.0317953)
Supplement: S2 Table — (PDF) [file pone.0317953.s002.pdf]

S2 Table The top-50 gene of DEGs

Gene ID	Gene name
ENSCHIG00000026834	GPS1
ENSCHIG00000001753	CCDC85B
ENSCHIG00000023501	EDL36312.1 mCG146085, partial [Mus musculus]
ENSCHIG00000016277	MRPL38
ENSCHIG00000025876	MMEL1
ENSCHIG00000009697	RFXANK
ENSCHIG00000026655	GDF1
ENSCHIG00000023781	ORC4
ENSCHIG00000014854	CTU2
ENSCHIG00000027060	XP_020741662.1 ribose-5-phosphate isomerase isoform X1 [Odocoileus virginianus texanus]
ENSCHIG00000007327	RAB3A
ENSCHIG00000015252	ARVCF
ENSCHIG00000024505	MFSD12
ENSCHIG00000023749	CD37
ENSCHIG00000015445	KAT5
ENSCHIG00000016266	-
ENSCHIG00000025635	ERCC1
ENSCHIG00000018001	EDL41015.1 mCG148442 [Mus musculus]
ENSCHIG00000024616	TATDN1
ENSCHIG00000009427	-
ENSCHIG00000019672	FAM166A
ENSCHIG00000023053	PHF1
ENSCHIG00000018628	DALRD3
ENSCHIG00000021644	CORO1A
ENSCHIG00000023795	TBC1D8
ENSCHIG00000009712	NP_001075822.1 sperm membrane protein-B [Oryctolagus cuniculus]
ENSCHIG00000006699	-
ENSCHIG00000011700	-
ENSCHIG00000014077	SLC5A2
ENSCHIG00000019537	-
ENSCHIG00000026127	SBNO2
ENSCHIG00000026216	XP_021524940.1 LOW QUALITY PROTEIN: seipin-like [Aotus nancymaae]
ENSCHIG00000020365	TECR
ENSCHIG00000018170	CLTB
ENSCHIG00000025310	PTK6
ENSCHIG00000021938	-
ENSCHIG00000013548	SPRYD3
ENSCHIG00000011388	-
ENSCHIG00000020973	ZSCAN29
ENSCHIG00000023525	IKBKG
ENSCHIG00000027270	XP_015313725.1 ETS domain-containing transcription factor ERF [Bos taurus]

ENSCHIG00000026314	PHYKPL
ENSCHIG00000023940	IDI1
ENSCHIG00000022403	NUP210L
ENSCHIG00000016675	BAH13183.1 unnamed protein product [Homo sapiens]
ENSCHIG00000021798	TSPOAP1
ENSCHIG00000020681	PIDD1
ENSCHIG00000025253	TP53BP2
ENSCHIG00000024359	SYDE1
