# Supplementary figures and images for: LIPG-mediated regulation of lipid deposition and proliferation in goat intramuscular preadipocytes involves the PPARα signaling pathway
Source: PLoS One. 2025 Feb 13;20(2):e0317953. doi: 10.1371/journal.pone.0317953 (PMC11825097; doi:10.1371/journal.pone.0317953)

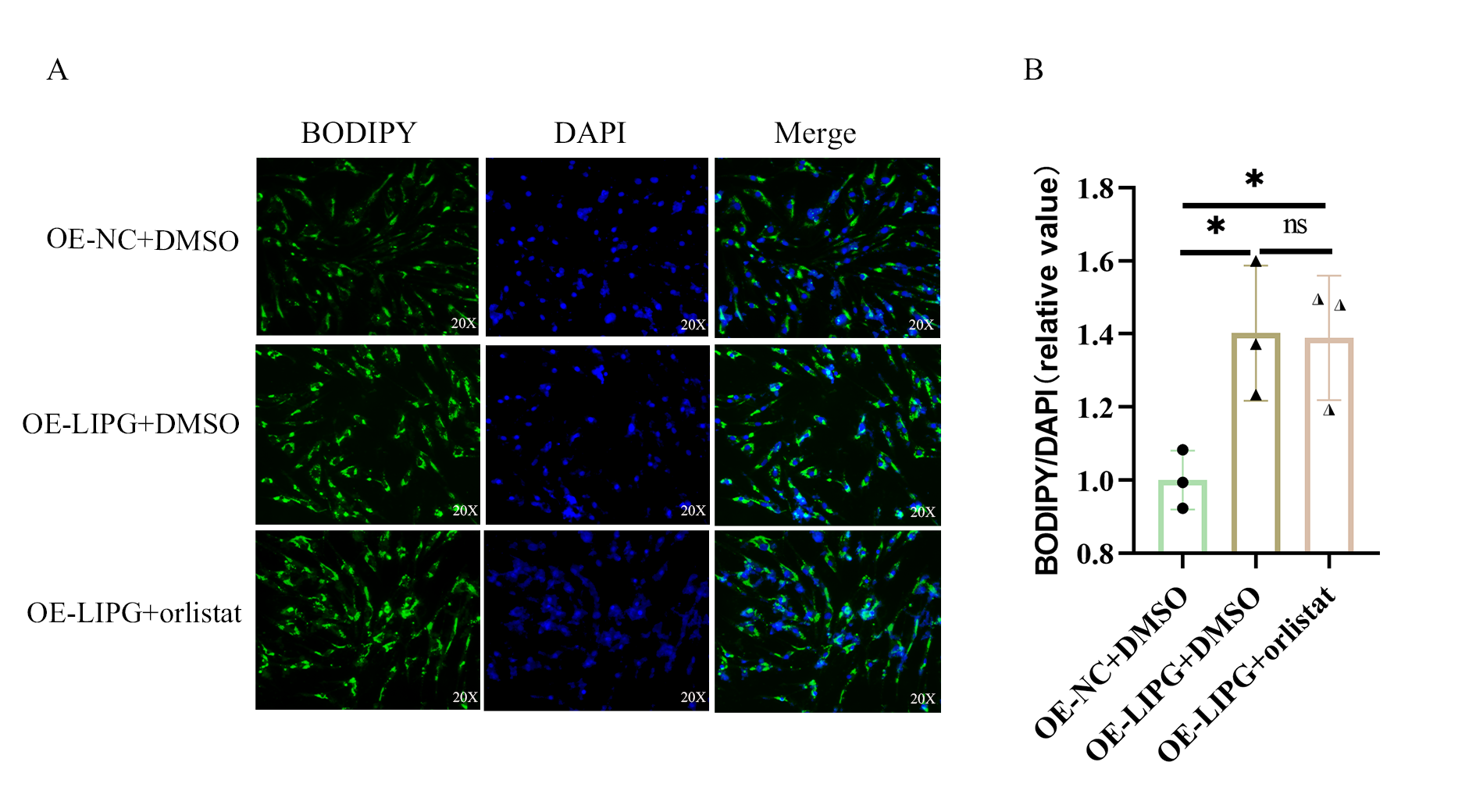

Supplement: S1 Fig — (TIF) [file pone.0317953.s003.tif]

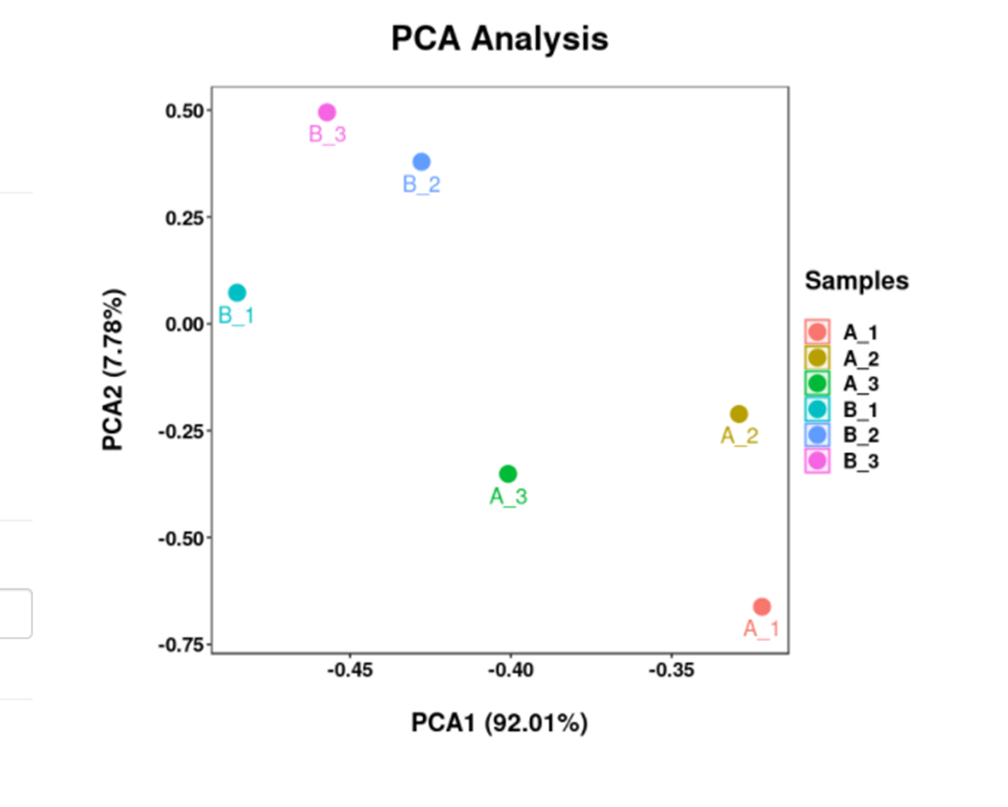

Supplement: S2 Fig — (TIF) [file pone.0317953.s004.tif]

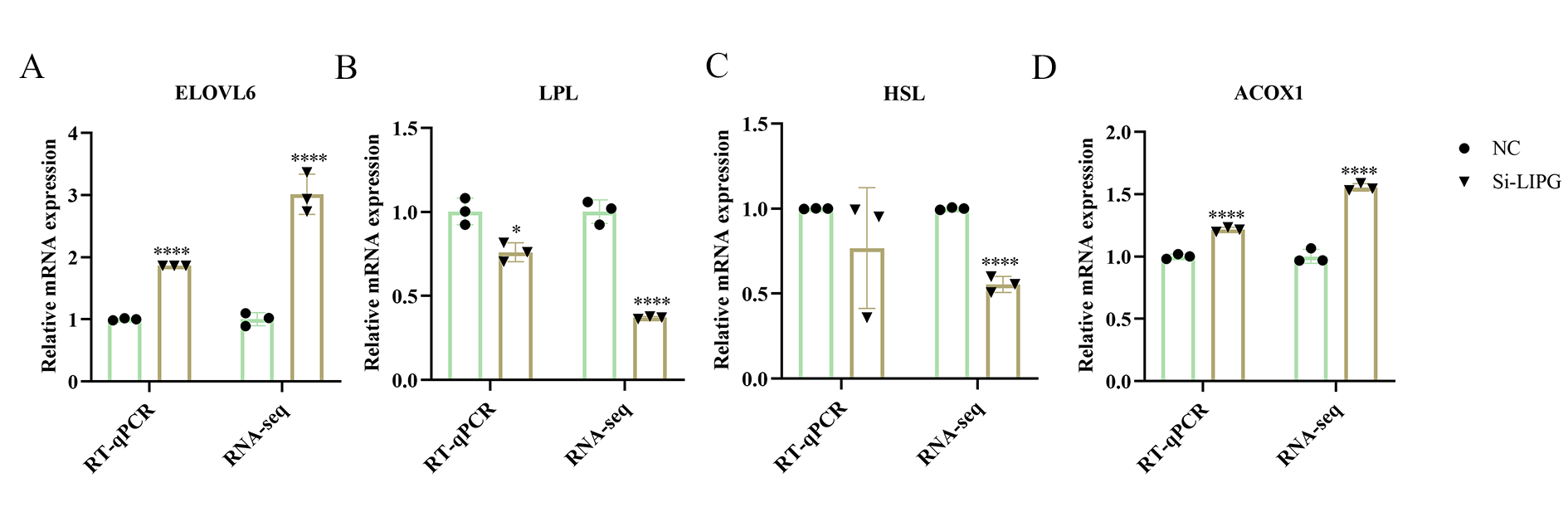

Supplement: S3 Fig — (TIF) [file pone.0317953.s005.tif]
