# Supplementary material for: LIPG-mediated regulation of lipid deposition and proliferation in goat intramuscular preadipocytes involves the PPARα signaling pathway
Source: PLoS One. 2025 Feb 13;20(2):e0317953. doi: 10.1371/journal.pone.0317953 (PMC11825097; doi:10.1371/journal.pone.0317953)
Supplement: S1 Raw Images — (PDF) [file pone.0317953.s006.pdf]

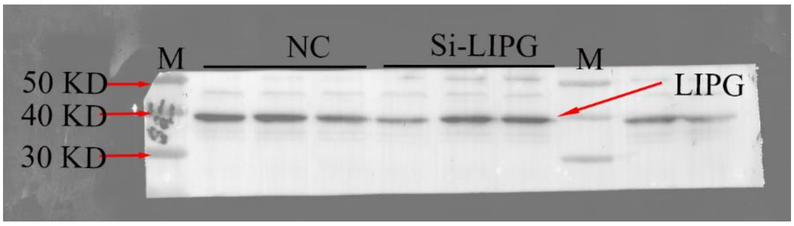

The original image of LIPG in Figure 2B. WB were used to detect the expression of LIPG in negative control groups (NC) and interfering LIPG gene groups (Si-LIPG).

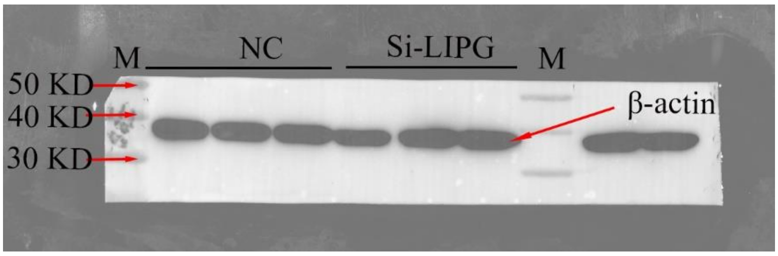

The original image of β-actin in Figure 2B. WB were used to detect the expression of β-actin in negative control groups (NC) and interfering LIPG gene groups (Si-LIPG).

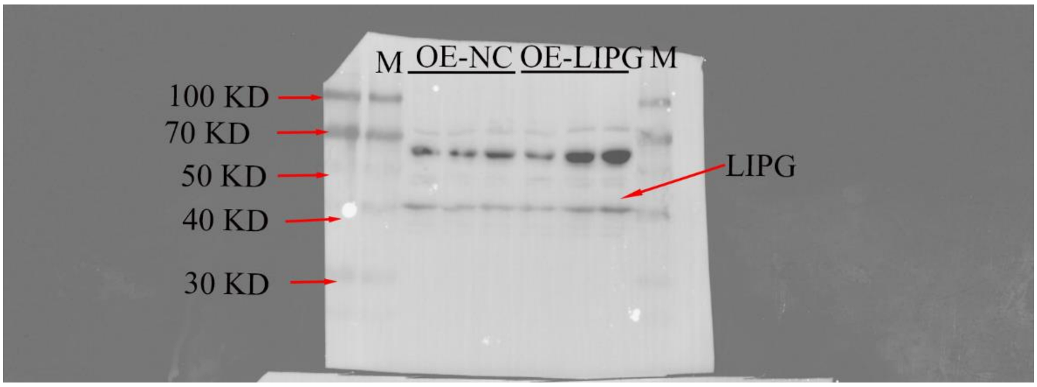

The original image of LIPG in Figure 4B. WB were used to detect the expression ofLIPG in negative control groups (OE-NC) and overexpression LIPG gene groups (OE-LIPG).

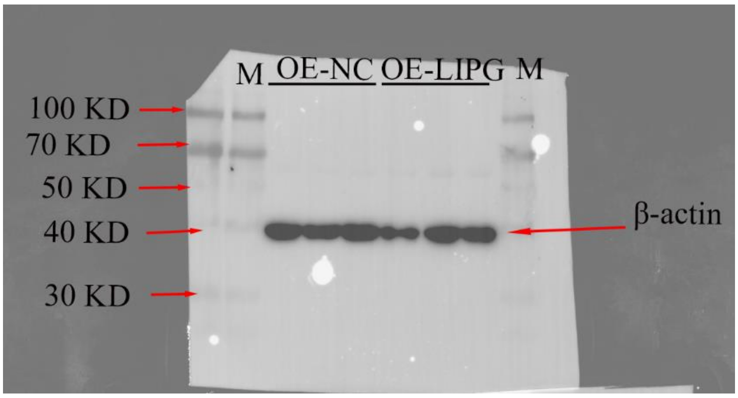

The original image of β-actin in Figure 4B. WB were used to detect the expression of β-actin in negative control groups (OE-NC) and overexpression LIPG gene groups (OE-LIPG).

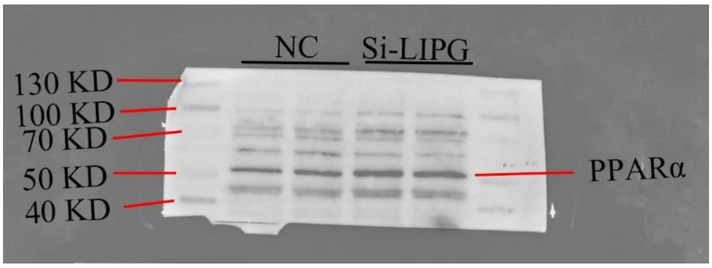

The original image of PPARα in Figure 6F. WB were used to detect the expression of PPARα in negative control groups (NC) and interfering LIPG gene groups (Si-LIPG).

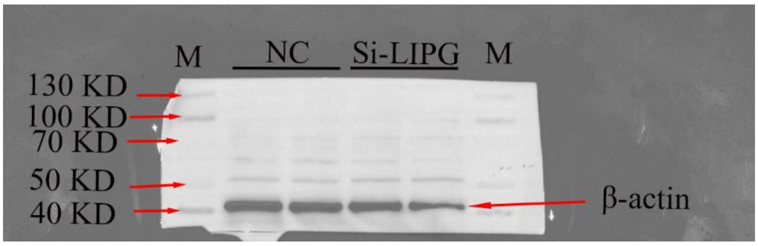

The original image of β-actin in Figure 6F. WB were used to detect the expression of β-actin in negative control groups (NC) and interfering LIPG gene groups (Si-LIPG).
