# Supplementary material for: LIPG-mediated regulation of lipid deposition and proliferation in goat intramuscular preadipocytes involves the PPARα signaling pathway
Source: PLoS One. 2025 Feb 13;20(2):e0317953. doi: 10.1371/journal.pone.0317953 (PMC11825097; doi:10.1371/journal.pone.0317953)
Supplement: Attachment — Submitted filename: Response to Reviewers.docx [file pone.0317953.s007.docx]

Response to Reviewers

Dear editors and reviewers:

Thanks for your comments concerning our manuscript entitled “LIPG promotes lipid deposition through PPARα signaling pathway in goat intramuscular preadipocyte” (PONE-D-23-34403). Those comments were valuable and very helpful. We have read the comments and the instructions for authors carefully. Revised portions are marked in red in the Revised Manuscript with Track Changes.

To editors

Q1: Please ensure that your manuscript meets PLOS ONE&#39;s style requirements, including those for file naming.

R1: Thank you for your comments, we have revised the manuscript as requested by PLOS ONE, including correctly named file names.

Q2: We noticed you have some minor occurrence of overlapping text with the following previous publication(s), which needs to be addressed:

- https://www.mdpi.com/1422-0067/24/17/13415

In your revision ensure you cite all your sources (including your own works), and quote or rephrase any duplicated text outside the methods section. Further consideration is dependent on these concerns being addressed.

R2: Thank you for your comments, we have redescribed the section of overlapping and correctly quote the relevant literature.

Q3: Thank you for stating the following financial disclosure: [Jiangjiang Zhu was supported by Sichuan Science and Technology Program (2021YFYZ0003)；Jiangjiang Zhu was supported by Zhejiang Science Technology Program (2022C04017)；Hua Xiang was supported by Sichuan Science and Technology Program (2022NSFC0082)；Yinggui Wang was supported by Graduate innovative scientific research project of Southwest Minzu University (ZD2022726)；].

Please state what role the funders took in the study. If the funders had no role, please state: &quot;&quot;The funders had no role in study design, data collection and analysis, decision to publish, or preparation of the manuscript.&quot;&quot;

If this statement is not correct you must amend it as needed.

Please include this amended Role of Funder statement in your cover letter; we will change the online submission form on your behalf.

R3: Thanks to your comments, we've added the Author Contributions and Author Contributions sections to the manuscript (line381-397).

Q4: PLOS ONE now requires that authors provide the original uncropped and unadjusted images underlying all blot or gel results reported in a submission’s figures or Supporting Information files. This policy and the journal’s other requirements for blot/gel reporting and figure preparation are described in detail at https://journals.plos.org/plosone/s/figures#loc-blot-and-gel-reporting-requirements and https://journals.plos.org/plosone/s/figures#loc-preparing-figures-from-image-files. When you submit your revised manuscript, please ensure that your figures adhere fully to these guidelines and provide the original underlying images for all blot or gel data reported in your submission. See the following link for instructions on providing the original image data: <https://journals.plos.org/plosone/s/figures#loc-original-images-for-blots-and-gels>.

In your cover letter, please note whether your blot/gel image data are in Supporting Information or posted at a public data repository, provide the repository URL if relevant, and provide specific details as to which raw blot/gel images, if any, are not available. Email us at plosone@plos.org if you have any questions.

R4: Thanks to your comments, we have added the original images of all the blot results in the supplementary material "S1_raw_images.pdf".

Q5: PLOS requires an ORCID iD for the corresponding author in Editorial Manager on papers submitted after December 6th, 2016. Please ensure that you have an ORCID iD and that it is validated in Editorial Manager. To do this, go to &lsquo;Update my Information&rsquo; (in the upper left-hand corner of the main menu), and click on the Fetch/Validate link next to the ORCID field. This will take you to the ORCID site and allow you to create a new iD or authenticate a pre-existing iD in Editorial Manager. Please see the following video for instructions on linking an ORCID iD to your Editorial Manager account: <https://www.youtube.com/watch?v=_xcclfuvtxQ&quot>;

R5: As requested, we have updated the ORID iD in Editorial Manager.

Q6: We note that Figure(s) 2B, 2D, 2G, 4B, 4D, 4G, 6B and 7D in your submission contain copyrighted images. All PLOS content is published under the Creative Commons Attribution License (CC BY 4.0), which means that the manuscript, images, and Supporting Information files will be freely available online, and any third party is permitted to access, download, copy, distribute, and use these materials in any way, even commercially, with proper attribution. For more information, see our copyright guidelines: <http://journals.plos.org/plosone/s/licenses-and-copyright>.

R6: All of these images were taken by the author, so there are no copyright issues.

To Reviewer #1

Q1: The manuscript is very poorly written with very poor English with a lot of grammar and syntax errors which make the reading very difficult.

R1: Thanks for your comments, we have made a lot of changes to the article in terms of language and writing.

Q1: Experimental details regarding Oil Red, BODIPY, and EDU staining are missing: the authors should provide information on the time frame between siRNA- and plasmid- transfection and staining with Oil Red, BODIPY, and EDU; were the cells incubated in a full medium with 10% FCS?

R1: Thanks for the comments. We have added a subsection to the Materials and Methods section to describe the time frame for transfection and subsequent experiments (line 113-122).

"After the end of transfection, the cell culture medium was replaced with induction medium (MEM/F12, 10% FBS, 1% Penicillin-Streptomycin Solution, 50 μmol/L oleic acid (Sigma, Tokyo, Japan))(31). After 48 hours, these cells were utilized for subsequent analyses including Oil Red O staining, BODIPY staining, triglyceride assay, EDU staining, and qPCR. "

The media used to culture the cells contained 10% FCS.

Q2: Page 6 lane 115; cells were treated with triglyceride lysate? What does this mean?

R2: We have changed the irregular description "triglyceride lysate" to the correct description "cell lysis" (line 139).

Q3: LIPG Western blot (WB) of untreated cells as well as of LIPG silenced and overexpressing cells should be performed and shown; WB of cell lysates and of heparin medium should be shown.

R3: We have assayed the protein content of LIPG in LIPG silenced and overexpressing cells using WB. These results were shown in Figure 2B-C and Figure 4B-C. (line 211, line 252)

Q4: It is not stated which statistical tests were used for the analyses.

R4: We have added a sentence in the statistical analysis section to describe the statistical tests that were used. (line 184-185)

"T-tests were used for statistical analysis when there were only two groups, and one-way ANOVA was used when there were three or more groups."

Q5: The authors should discuss why LPL is upregulated after LIPG overexpression. Previous studies have shown upregulation of EL in WAT of LPL-KO mice.

R5: Thanks for your comments, we've added this section to the discussion section. (line 352-357)

"We are puzzled that overexpression of LIPG increased LPL expression in goat intramuscular precursor adipocytes. Previous studies have shown that Lipg-/- mice have higher levels of LPL expression than WT mice, and it has been demonstrated that LIPG and LPL collaborate to promote efficient triglyceride (TG)-rich lipoproteins (TRLs) lipolysis (36). The differences between our results and the reported in mice may be due to cell-individual differences and ethnic differences between mice and goats. Obtaining knock-out goats for in vivo studies of LIPG gene function related to IMF deposition is likely not feasible due to the high cost and concern for animal welfare."

Q6: Description of Fig. 7 is confusing and something is not correct. The authors show the impact of LIPG silencing on PPARa, then the impact of overexpression on lipid accumulation (BODIPY) and also graph with EDU results, but state in the legend it is BODIPY. Fig. 7F shows upregulated ATGL after LIPG overexpression which is contradictory to Figs 3 and 5.

R6: Thank you for your recommendation, we have reorganized Figure 7 and have removed the inappropriate description.

Q7: Page 13, lane 305: It is not correct that LIPG was identified first in endothelial-type adipocytes; it was in HUVEC.

R7: Thank you very much for pointing out the inprecision in our writing. We have changed "endothelial type adipocytes" to "Human Umbilical Vein Endothelial Cells (HUVEC)". (line 339-340)

Q8: Page 14, lane 331: The reference 48 is wrong; this paper is on HL and not on LIPG and PPARa.

R8: We have corrected the references (Ahmed in drugi, 2006). (line 369)

References:

Ahmed, W., Orasanu, G., Nehra, V., Asatryan, L., Rader, D. J., Ziouzenkova, O. in Plutzky, J. (2006). High-density lipoprotein hydrolysis by endothelial lipase activates PPARalpha: a candidate mechanism for high-density lipoprotein-mediated repression of leukocyte adhesion. Circulation Reserch, 98(4), 490-498. https://doi.org/10.1161/01.RES.0000205846.46812

To Reviewer #2

Q1: What was the rationale to study the role of LIPG in goat specifically? Is it indeed as mentioned on page 3 to improve goat meat quality? I bet there will be better targets than LIPG and alternative methods to do this. Please explain in more detail.

R1: Thanks for the comments. We also believe that there will be better targets than LIPG and alternative methods to improve the quality of goat meat. We studied LIPG in order to reveal the regulatory effects of LIPG on lipid deposition in goat intramuscular preadipocytes, and these efforts will help to expand the regulatory network of intramuscular fat (IMF) and provide a theoretical basis for improving meat quality. As our previous formulation could be misleading to readers, we have changed it to "Conclusively, these findings may contribute to our understanding of the lipid metabolism regulation network and provide a theoretical basis for using genetic breeding techniques to improve the quality of goat meat". (line 70-72)

Q2: How do the results in the current paper compare to data available from, for instance, LIPG knockout mice? Do they show similar effects on adipose tissue? This should be discussed in the discussion.

R2: Thank you for your comment, we have added a section to the discussion to compare with current paper. (Line 345-346, 352-355, 367-369)

Q3: I’m completely lost in the way the authors culture the cells and performed their experiments. Firstly, how are they sure that they collected the pre-adipocytes from the big piece of muscle they collected from goats? I’d advise to show some typical pre-adipocyte markers before and directly after isolation form the muscle as well as after the differentiation.

R3: Thank you for your comment! Previous studies have described the protocol to Isolated intramuscular preadipocytes and myoblasts from dorsal longest muscle. In simple terms, cells are differentiated according to the adhesion time, the intramuscular preadipocytes attach to the wall in 0-2h, and the muscle cells attach to the wall in 2-72h(Qiu in drugi, 2018).Therefore, we collected preadipocytes by removing the culture medium and nonadherent cells at 2h. At the same time, we have added a description of how preadipocytes are collected from dorsal longest muscle in Materials and Methods. (Line 93-95)

"Previous studies have shown that preadipocytes were adhere at 0-2 h and myogenic cells at 2-74 h (30). Intramuscular preadipocytes were isolated by removing nonadherent cells from the supernatant after 2 h. "

A number of adipocyte markers have been examined in our previous studies at different times of differentiation(Tian in drugi, 2022).

References:

Qiu, K., Zhang, X., Wang, L., Jiao, N., Xu, D. in Yin, J. (2018). Protein Expression Landscape Defines the Differentiation Potential Specificity of Adipogenic and Myogenic Precursors in the Skeletal Muscle. Journal of Proteome Research, 17(11), 3853-3865. https://doi.org/10.1021/acs.jproteome.8b00530

Tian, W., Xiang, H., Li, Q., Wang, Y., Zhu, J., Lin, Y. in Jois, M. (2022). Oleic acid, independent of insulin, promotes differentiation of goat primary preadipocytes. Animal Production Science, 63(2), 113-119. https://doi.org/10.1071/an21155

Q4: Secondly, is adding oleic acid enough to get adipocytes or are the cultured cells in the end still pre-adipocytes? Looking at the figures I get the impression that these cells are still the latter. So why use this protocol and not more established ones like adding a PPARgamma agonist and insulin?

R4: Our previous results showed that the cells still had good differentiation ability after 48h induction by oleic acid. Our previous results showed that preadipocytes could self-differentiate for more than one week under natural induction, although this process may be shortened under oleic acid induction, but morphologically, it is true that preadipocytes have a fibrous morphology rather than mature adipocyte morphology. In addition, we also compared two protocols, the addition of insulin and the addition of oleic acid, and found that oleic acid induced better differentiation of goat preadipocytes (Tian in drugi, 2022).

Reference:

Tian, W., Xiang, H., Li, Q., Wang, Y., Zhu, J., Lin, Y. in Jois, M. (2022). Oleic acid, independent of insulin, promotes differentiation of goat primary preadipocytes. Animal Production Science, 63(2), 113-119. https://doi.org/10.1071/an21155

Q5: Can the authors also provide data on whether the differentiation worked by for instance showing bona fide adipogenesis as well as proliferation markers at various time-points?

R5: Our previous studies have detected lipid marker genes at different time points, indicating that intramuscular precursor adipocytes have good lipid formation ability during differentiation (Tian in drugi, 2022). We added the expression profiles of proliferation marker genes at different time points to the supplementary materials, indicating that the proliferation capacity of preadipocytes still exists in spite of the decreased proliferative ability during differentiation. In addition, we also added this part in the paper. (Line 219-220)

"In the process of inducing the intramuscular preadipocytes to differentiate into adipocytes, the proliferation capacity of the cells was decreased but still existed (S2 Fig)."

Reference:

Tian, W., Xiang, H., Li, Q., Wang, Y., Zhu, J., Lin, Y. in Jois, M. (2022). Oleic acid, independent of insulin, promotes differentiation of goat primary preadipocytes. Animal Production Science, 63(2), 113-119. <https://doi.org/10.1071/an21155>

Q6: Thirdly, the information on when the exact experiments were performed are scarce. Please provide more details on this. When exactly were the measurements performed after plasmid or siRNA transfection? If this indeed for a short while, can we truly call it an effect of LIPG on proliferation? I think we might need more tiem for that, i.e. by knocking down LIPG by shRNA.

R6: We added the exact times of plasmid transfection, siRNA transfection, and subsequent experiments in line 120.

We are confident that siRNA is consistently inhibiting LIPG expression within 48 h, as significant interference efficiency was detected by us at 48 h in line 210.

Therefore, we truly call it an effect of LIPG on proliferation.

Q7: The information on statistical analysis is scarce. What kind of test was used, how large were the numbers? Moreover, the results with WY-14643 and the overexpression need to be analyzed with a two-way ANOVA but it looks like the authors performed a simple t-test.

R7: Thanks to your reminder, we added descriptions of the number of biological replications and statistical methods. (line 184-187)

"T-tests were used for statistical analysis when there were only two groups, and one-way ANOVA was used when there were three or more groups. The number of all biological replicates in the experiment was three (n=3). Significance was indicated using "*" p<0.05, "**" p<0.01, "***" p<0.001, "****" p<0.0001."

Q8: Figure 1C shows data on the LIPG expression in various goat tissues but there is no information in the material and methods section on how these tissues were collected.

R8: Thank you for the reminder, we have added a paragraph in Materials and Methods to explain the process of collecting tissue. (Line 80-82)

"Three 12-month-old Jianzhou goat were randomly selected from Sichuan Jianyang Dageda Animal Husbandry. Heart, liver, spleen, kidney, longest dorsal muscle, subcutaneous fat, and small intestine were taken quickly after euthanasia, washed using DEPC water and frozen in liquid nitrogen for preparation."

Q9: Although the authors describe in line 191-192 that there was a suppressed intracellular TAG after LIPG knockdown this is actually not the case according to Figure 2F since a don’t see significant asterisk.

R9: We believe that the downregulation of intracellular TAG by LIPG knockdown is due to its downward trend, which is consistent with the change trend of lipid droplet content. As for the NC group and the processing group TAG is not significant, it is possible that the number of samples (n=3) and the sensitivity of the test kit can lead to large standard deviations, which can affect the significance analysis.

Q10: Looking at figures 2B and 4B get the impression that the cells are still pre-adipocytes with relatively low lipid content but I cannot completely be sure since a ‘simple’ picture showing the cells in culture is missing.

R10: Thanks for your comments, we have added pictures where you can see the cell morphology. (Fig 2D, Fig 4D)

Q11; In addition, the data in Figures 2G and 4G show that the proliferation is very low in the control situation making the effects of LIPG knockdown on this rather questionable. Is this truly an effect?

R12: The relatively low positive labeling in the NC group observed by Figures 2G and 4G may be due to the photographic process. It is true that knockdown or overexpression of LIPG leads to proliferation alteration. For data reliability, we reran the experiment and obtained the same results as before. At the same time, we improved the photographic method and replaced 2G and 4G with the latest experimental results.

Q13: Is the very small induction of the FADS1 mRNA expression shown in Figure 3B truly an effect? I doubt it with such a small difference.

R13: In Figure 3B, the expression of FADS1 mRNA increased by 10%, and the small change seen in the figure may be due to the excessive value of the horizontal coordinate. The expression of FADS1 mRNA with significant changes was analyzed by SPASS.

Q14: I’m puzzled by the fact that the si-LIPG treated cells have a higher PPARa level (see Figure 7A-C) but at the same time have reduced mRNA expression of typical PPARa target genes such as CPT1a and ACOX1). Please explain in the discussion why this might linked. Or is something else going on?

R14: In our experiments, interfering with LIPG increased PPARα mRNA expression, while mRNA expression of ACOX1 was also increased. We repeated the experiment 3 times and obtained the same results that interfering LIPG decreased the mRNA expression level of CPT1. CPT1 is regulated by AMPK, MAPK and other signaling pathways in addition to PPARα. Therefore, the reduction of CPT1 mRNA expression level may be regulated by other signaling pathways or regulatory factors. We also explain this in the discussion section.

"CPT1 may be regulated by PPARa, AMPK, MAPK and other signaling pathways(He in drugi, 2021; Vandanmagsar in drugi, 2016). Knockdown of LIPG increased the expression level of PPARa but decreased the expression level of CPT1A and CPT1B, the target genes of PPARa, suggesting that its LIPG regulates lipid deposition in goat intramuscular preadipose partly through the PPARa signaling pathway."

References:

He, W., Gao, M., Yang, R., Zhao, Z., Mi, J., Sun, H., . Fang, X. (2021). The effect of CPT1B gene on lipid metabolism and its polymorphism analysis in Chinese Simmental cattle. Anim Biotechnol, 1-13. https://doi.org/10.1080/10495398.2021.1904966

Vandanmagsar, B., Warfel, J. D., Wicks, S. E., Ghosh, S., Salbaum, J. M., Burk, D., . Mynatt, R. L. (2016). Impaired Mitochondrial Fat Oxidation Induces FGF21 in Muscle. Cell Rep, 15(8), 1686-1699. https://doi.org/10.1016/j.celrep.2016.04.057

Q15: It would be great of the authors could provide more information on the genes that are significantly differently expressed between si-LIPG treated and control cells. Why not provide at least a list of the top-50 up- and down-regulated genes? Now I’m only getting very curious which gene is depicted in the top right in Figure 6A. It can’t be LIPG since this gene is upregulated.

R15: We have added the top50 differential genes in the S2 Table. The most significantly upregulated was the G Protein Pathway Suppressor 1 (GPS1) gene, followed by the Coiled-Coil Domain Containing 85B (Delta-Interacting Protein A) CCDC85B gene. (Line 291-293)

Q16: In lines 313-314 the authors state that the LIPG knockdown is assumed to result in elongation of fatty acids. Why do they think so? Is this because of the increased Elovl6 mRNA expression? Why didn’t the authors analyze the fatty acid composition to check this?

R16: Thanks for your comments, we propose this hypothesis based on changes in mRNA levels and in RNA-seq of Elovl6. We realized that analysis of fatty acid composition would be more beneficial to explain the role of LIPG, but limited by the experimental equipment and the cost of the experiment, we are unable to analyze the fatty acids further. At the same time, we also realize that the available evidence is insufficient to support our view. Therefore, we deleted this part from the article.

Q17: In lines 316-318 the authors state that the expression of ATGL was ‘was increased extremely significantly increased by LIPG silencing’. Apart from the strange sentence, I don’t think you can call a 10% extremely! Aren’t the authors over interpreting the mRNA data here?

R17: We are very sorry that our loose description misinterpreted you, but the 17% increase in ATGL after knocking down LIPG was a significant change by t-test. In order not to be misleading, we have deleted the loose description "extremely significantly increased".

To Reviewer #3

Q1: The authors conclude that LIPG is acting through PPAR alpha, but the data don’t strongly support this. Yes, a PPAR alpha agonist normalized some of the geneexpression changes, but it did not normalize increased lipid accumulation or cell proliferation (as shown in Fig. 7 D and E).

R1: We agree with the suggestions made by the reviewers. At the same time, we have to admit that LIPG does not work only through PPARα signaling pathway. Therefore, we changed the title of the paper to "LIPG regulates lipid deposition partly through PPARα signaling pathway in goat intramuscular preadipocytes ". (Line 4)

Q2: The authors show a lot of gene expression data, but these data don’t indicate a clear mechanism (for example, ATGL and HSL go different directions) and there is no follow up (western blots, mechanistic assays) so these expression data don’t provide very much insight. Moreover, there is some lack of consistency. In figure 5D, ATGL expression decreases with LIPG overexpression, but in Figure 7F, ATGL expression increases 3 fold with LIPG overexpression.

R2: Thank you for your comments, we did not perform WB on lipid metabolism-related genes due to the lack of antibodies. The changes in mRNA levels are also representative of trends to a certain extent, so we have rediscussed this part. In addition, we re-examined the mRNA expression of ATGL and determined that overexpression of LIPG reduced ATGL expression. (Line 347-360)

"Knockdown LIPG reduced de novo fatty acid synthesis by down-regulating the expression of FASN, a key enzyme in de novo fatty acid synthesis (41). It also reduced lipid droplet content by increasing ATGL to lipolysis ATG into diacylglycerol (DAG) and FAs (45, 46). However, HSL, the main decomposing agent of DAG (45), was not reduced. It is possible that the lipolysis product FAs is involved in the regulation of HSL as a signaling molecule. Taken together, the promotion of cellular lipid deposition by LIPG observed in goat intramuscular preadipocyte may be achieved by increasing de novo fatty acid synthesis and decreasing lipolysis."

Q3: It is not clear if any of the changes seen with LIPG overexpression actually require LIPG activity. This could easily be tested with Tetrahydrolipstatin (orlistat).

R3: Based on the reviewers' comments, we added orlistat to cells overexpressing LIPG and found that orlistat could not restore the increased lipid deposition by overexpression of LIPG. At the same time, we also added this part to the manuscript. (Line 260-265)

"To investigate the role of LIPG activity in promoting lipid deposition induced by overexpression of LIPG, we added orlistat to intramuscular preadipocytes overexpressing the LIPG gene. Addition of orlistat did not restore the increase in lipid deposition caused by overexpression of LIPG (S1 Fig), suggesting that LIPG-promoted lipid deposition does not act through LIPG activity."

Q4: For both overexpression and knockdown studies, the assays measuring actual TG levels, don’t show a difference between control cells and overexpression or knockdown cells.

R4: Thank you for the reminder that we have analyzed the triglyceride data for significance and labeled it in Figure 2H and Figure 4H.

Q5: In LIPG overexpression assays, LIPG expression is increased over a thousand fold. This seems to indicate that there is very little LIPG expression normally in these cells, which might suggest LIPG is not strongly relevant to adipogenesis in these cells.

R5: Our RT-qPCR results showed that LIPG expression was close to that of the internal reference gene UXT (stable and highly expressed in goat intramuscular precursor adipocytes (Xu Q in drugi, 2018)), so a thousand fold may be a result of high vector expression efficiency or high transfection efficiency.

Xu Q, Lin S, Zhu J J, Y Wang in Lin, Y. (2018). The expression stability analysis of reference genes in the process of goat intramuscular preadipocytes differentiation in goat. Acta Veterinaria et Zootechnica Sinica, 49(5), 907-918.

Q6: Overall the writing needs editing for clarity and accuracy.

R6: Thank you for your comments, we have made a lot of revisions to the article in writing.

Q7: The authors need to indicate what statistical tests they used to determine p values.

R7: Thanks to your comments, we have added the statistical tests to the article.

"T-tests were used for statistical analysis when there were only two groups, and one-way ANOVA was used when there were three or more groups. The number of all biological replicates in the experiment was three (n=3). Significance was indicated using "*" p<0.05, "**" p<0.01, "***" p<0.001, "****" p<0.0001." (Line 184-178)

We sincerely appreciate for your comments, and hope the responses will clarify the problems.

Best regards

Yinggui Wang
