# Supplementary material for: LIPG-mediated regulation of lipid deposition and proliferation in goat intramuscular preadipocytes involves the PPARα signaling pathway
Source: PLoS One. 2025 Feb 13;20(2):e0317953. doi: 10.1371/journal.pone.0317953 (PMC11825097; doi:10.1371/journal.pone.0317953)
Supplement: Attachment — Submitted filename: Response_to_Reviewers_auresp_2.docx [file pone.0317953.s008.docx]

Response to Reviewers

Dear editors and reviewers:

Thanks for your comments concerning our manuscript entitled “LIPG promotes lipid deposition through PPARα signaling pathway in goat intramuscular preadipocyte” (PONE-D-23-34403). Those comments were valuable and very helpful. We have read the comments and the instructions for authors carefully. Revised portions are marked in red in the Revised Manuscript with Track Changes.

To Reviewer 1

Q1: Lane 219: ..the proliferation capacity.. (S2 Fig.). This Fig shows lipid accumulation (BODIPY) but not proliferation.

R1: Thank you for pointing out this discrepancy. We have corrected the text to accurately reflect the content of the figure. In the process of inducing the intramuscular preadipocytes to differentiate into adipocytes, the proliferation capacity of the cells was decreased by EDU staining (Fig 2I-2J).” We appreciate your attention to detail.

Q2: Lane 262:Addition of orlistat did not restore… I would suggest here and in the other parts of the manuscript replacing restore with: attenuate or inhibit or prevent or decrease.

R2: Thank you for your helpful suggestion. We have replaced "restore" with "attenuate" in the revised sentence, which now reads: " Addition of orlistat did not attenuate the increase in lipid deposition caused by overexpression of LIPG (S2 Fig)".

Q3: The authors did not describe in the methods and figure legend the experimental details regarding orlistat treatment (concentration, duration of pre-treatment etc.).

R3: Thank you for pointing this out. We have added the relevant experimental details regarding the orlistat treatment to the Materials and Methods section. The revised text now reads: " Transfected cells were treated with 20 μM orlistat (dissolved in DMSO) and cultured for an additional 48 h." We hope this clarifies the methodology used in our experiments.

Q4: The authors should test whether the applied amounts/concentrations of orlistat indeed attenuate LIPG activity. If orlistat indeed had no impact on LIPG-overexpression-induced lipid accumulation (which is surprising and very interesting) the authors should try to provide an explanation how LIPG for example by its bridging function or some other mechanisms, other than by provision of FFA, affects PPAR activity and lipid accumulation.

R4: Thank you for this valuable recommendation. We understand the importance of confirming whether the applied concentration of orlistat attenuates LIPG activity. However, due to current limitations in our laboratory facilities, we are unable to conduct this specific experiment at this time. We have, however, added a discussion of this limitation and its potential implications in the Discussion section to acknowledge the need for future investigation:

"However, our data from the orlistat experiment suggest that lipid accumulation changes associated with LIPG overexpression may not be solely dependent on its enzymatic activity, as orlistat treatment did not reverse the increased lipid accumulation. This raises the possibility that LIPG could also regulate lipid deposition through non-enzymatic mechanisms, particularly in goat intramuscular preadipocytes. While these findings contribute to our understanding, further experimental validation is needed to clarify the precise mechanisms—both enzymatic and potential non-enzymatic—through which LIPG regulates lipid metabolism and the PPARα pathway."

We hope this addresses your concern and we look forward to future investigations on this topic.

Q5: This part should be more carefully and precisely described. It is not correct as presently presented that OE-LIPG+WY14643 groups had more lipid droplets than OE-LIPG…..

It is not correct that PPARa can partially rescue (better: decrease)…The Figure shows that PPARa agonist significantly decreased lipid accumulation to the levels in the corresponding controls.

R5: Thank you very much for your careful review and valuable comments. We apologize for the errors and any confusion caused by these inaccuracies in the original manuscript. We have now thoroughly revised the relevant sections to ensure clarity and accuracy.

We appreciate your pointing out the inaccurate description of lipid droplet accumulation in the OE-LIPG+WY14643 groups. We have corrected this in the revised manuscript to more precisely reflect that the PPARα agonist significantly decreased lipid accumulation to levels similar to the corresponding controls. (Line 319-320)

Furthermore, to provide a more accurate description, we have amended the phrasing regarding the “partially rescue” effect of PPARα. Instead, we now describe that the PPARα agonist significantly decreased lipid accumulation, not partially rescuing the effect.To better reflect these changes and the focus of our findings, we have also modified the title of the manuscript to: “LIPG-mediated regulation of lipid deposition and proliferation in goat intramuscular preadipocytes involves the PPARα signaling pathway.”Thank you again for your constructive feedback.

Q6: There is a discrepancy between Figure legend and the text regarding labeling of the subfigures. (Text 7A-F; legend A-C).

R6: Thank you for pointing out the discrepancy between the figure legend and the text regarding the labeling of the subfigures. We have carefully reviewed both the figure and the legend and made the necessary corrections. Specifically, we have updated the legend to label the subfigures as 7A-7F, which now matches the description in the text. We believe these changes address the issue and enhance the accuracy of the manuscript. Thank you again for your valuable feedback.

Q7: Lane 355: Not ethnic, but species specific differences.

R7: Thank you for your valuable comment. We have corrected the text to reflect that the differences observed are not ethnic, but rather species-specific.

To Reviewer 1

Q1: The authors continue to assert that LIPG regulates cell proliferation and lipid deposition at least in part through PPAR alpha, but the data still don’t strongly support this. Yes, a PPAR alpha agonist normalized some of the gene expression changes, but data for cell proliferation is not shown and the agonist did not normalize increased lipid accumulation. True total lipid accumulation is lower with the agonist but it is lower both with and without LIPG overexpression and the induction by LIPG overexpression is similar suggesting that the change in lipid accumulation is independent of PPAR.

R1: Thank you very much for your insightful comments, which have prompted us to reconsider the scope of our title and clarify the role of the PPARα signaling pathway in LIPG-mediated regulation. In response to your feedback, we have adjusted the title to better reflect our findings that, while PPARα signaling is involved, it does not fully account for the effects of LIPG on lipid deposition and cell proliferation in goat intramuscular preadipocytes. The revised title is as follows:

“LIPG-mediated regulation of lipid deposition and proliferation in goat intramuscular preadipocytes involves the PPARα signaling pathway”

This updated title emphasizes that PPARα signaling is one pathway among potentially several through which LIPG influences lipid metabolism and cell proliferation, aligning more closely with our data. We acknowledge that while the PPARα agonist (WY14643) reduced lipid accumulation to some extent in the LIPG-overexpressing cells, it did not fully normalize the increase in lipid deposition, suggesting that additional signaling mechanisms may also contribute to LIPG’s regulatory role.

Q2: In the original submission, the authors do not perform any experiments to address the mechanism whereby endothelial lipase may induce lipid accumulation. In the revised experiment the authors perform an experiment with orlistat, which should inhibit the enzymatic activity of endothelial lipase. Unfortunately the authors do not perform controls to show that the orlistat was effective (experiments demonstrating a change in lipase activity with LIPG overexpression and with orlistat treatment should be included). Moreover if the orlistat was effective the authors show that there is no change in lipid accumulation (cell proliferation was not tested). This would suggest that the effect on lipid accumulation is not dependent on the enzymatic activity of endothelial lipase. The authors state this, at yet their hypothesis at the conclusion of their discussion is that EL-mediated hydrolysis liberates fatty acids that act on PPAR. Lipolysis requires endothelial lipase activity so how could this happen if endothelial lipase activity is not required?

R2: Thank you for your insightful and constructive feedback. We greatly appreciate your careful analysis of our study, particularly your comments regarding the orlistat experiment and its implications for LIPG's enzymatic activity. In response to your concerns, we acknowledge that orlistat treatment did not reverse the increase in lipid accumulation induced by LIPG overexpression. This observation suggests that the regulation of lipid accumulation by LIPG may not be entirely dependent on its enzymatic activity. As we discussed in the revised manuscript, we now hypothesize that LIPG might also exert its regulatory effects through non-enzymatic mechanisms in addition to its known enzymatic function. This aspect of LIPG’s role requires further investigation, and we plan to explore these non-enzymatic pathways in future studies.

We also recognize that our study did not provide direct evidence to confirm that orlistat effectively inhibited LIPG enzymatic activity. We agree with your suggestion that future experiments should include controls demonstrating a change in lipase activity with LIPG overexpression and orlistat treatment. This would strengthen our conclusions and provide clearer evidence regarding the enzymatic versus non-enzymatic contributions of LIPG. Despite these limitations, our current findings suggest that LIPG regulates lipid deposition through multiple pathways, potentially including both enzymatic and non-enzymatic mechanisms. We have emphasized this point in the revised discussion, acknowledging the need for further experimental validation. Once again, thank you for your valuable input, which has helped us clarify our hypotheses and the direction of future research. We are committed to addressing these issues in subsequent experiments.

Q3: As before, the authors show a lot of gene expression data, but these data don’t indicate a clear mechanism (for example, ATGL and HSL go different directions) and there is no follow up (western blots, mechanistic assays) so these expression data don’t provide very much insight. Moreover, to address the lack of consistency with the ATGL expression data in the original submission, they have simply removed the ATGL expression data from figure 7 without adequate explanation.

R3: Thank you for your valuable feedback. We appreciate your concerns regarding the gene expression data and the lack of mechanistic follow-up, as well as the issue with the ATGL expression data in Figure 7. In response to your first point, we understand that while gene expression data provide valuable insights into the transcriptional regulation of lipid metabolism, they alone do not fully elucidate the underlying mechanisms. We agree that further validation through Western blot analysis and mechanistic assays would significantly strengthen the conclusions of our study. Unfortunately, due to experimental constraints, we were unable to perform these additional experiments in the current version of the manuscript. However, we acknowledge the importance of these follow-up experiments and plan to incorporate them in future studies to provide a more comprehensive understanding of LIPG’s role in lipid accumulation and its impact on gene regulation. Regarding the issue with ATGL expression data, we recognize that the inconsistency in the ATGL expression data between our original submission and the revised manuscript may have raised concerns. In the revised manuscript, we made the decision to remove the ATGL data from Figure 7 due to its lack of consistency with the other experimental results and to avoid any confusion. We should have provided a more thorough explanation for this change in the manuscript. To address this, we have revised the text in the discussion to clarify that the ATGL data were removed due to inconsistencies with the overall findings and we acknowledge the need for further investigation into its potential role in lipid metabolism in the context of LIPG regulation. We believe that the data we have presented, along with the modifications in the manuscript, still provide valuable insights into the role of LIPG in regulating lipid deposition through its effects on various lipid metabolism-related genes and the PPARα signaling pathway. We hope that future experiments, including Western blotting and other mechanistic assays, will help to clarify these findings in more detail. Thank you again for your thoughtful suggestions. We will take these points into consideration for the next phase of our research and in preparing future revisions of the manuscript

Q4: For both overexpression and knockdown studies, the assays measuring actual TG levels, don’t show a difference between control cells and overexpression or knockdown cells. There may be a non-statistically significant trend, but the data does not match the BODIPY or Oil Red O data.

R4: We appreciate your thoughtful review of our manuscript and your comments regarding the discrepancy between the TG levels and the BODIPY and Oil Red O data.

We would like to clarify that, although the TG levels did not show statistically significant differences between control, overexpression, and knockdown cells, the trend observed was consistent with the BODIPY and Oil Red O staining results. Specifically, overexpression of LIPG led to an increase in TG levels, while knockdown resulted in a decrease, mirroring the pattern observed in lipid accumulation with BODIPY and Oil Red O staining. This consistency suggests that, despite the lack of statistical significance in the TG assay, the trend supports our findings regarding the regulation of lipid deposition by LIPG. We hope this explanation helps address the concerns raised, and we appreciate your constructive input as it has guided us in clarifying this point in our revised manuscript.

Q5: Graphs should show individual points not just bars and error bars.

R5: Thank you for your valuable feedback. In response to your suggestion, we have revised the graphs to include individual data points alongside the bars and error bars. This adjustment allows for a clearer representation of data distribution and variability, providing a more detailed view for the readers. We hope this modification meets your expectations and enhances the clarity and quality of our presentation.

Q6: The last sentence of the abstract is repeated.

R6: Thank you for pointing this out. We have revised the abstract to eliminate the repetition in the last sentence. The revised version now reads more clearly and concisely. We appreciate your attention to this detail.

Q7: When the authors perform experiments with PPAR agonists they often use the terms “restore” or “rescued” when I think what they mean is “reversed”.

R7: Thank you for your valuable comment. We appreciate your suggestion. Upon review, we agree that the terms “restore” or “rescued” may not be the most appropriate in this context. We have revised the manuscript to use the term "reversed" where applicable, to better reflect the intended meaning of the experiments with PPAR agonists. We believe this change clarifies the results more accurately. Thank you again for helping improve the clarity of our manuscript.

Q8: Authors mention that LIPG was identified at major differential gene between fat-tailed and thin-railed sheep. Do they mean thin-tailed, and though they say they have different lipid storage patterns they don’t explain what those are.

R8: Thank you for your comment. We apologize for the confusion caused by the incorrect spelling and have corrected it to "thin-tailed" as you suggested. We acknowledge that the reference cited (27) does not explicitly describe the specific lipid storage patterns in fat-tailed and thin-tailed sheep. To clarify, our intention was to convey that previous studies have identified LIPG as a differentially expressed gene between these two types of sheep, which are known to exhibit differences in their lipid storage characteristics.

Q9: The authors state, “Our findings are similar to previous reports that interfered LIPG inhibits lipid deposition and cell proliferation in goat intramuscular preadipocytes.” But none of the references that follow (41, 42, and 43) examined LIPG or used goat intramuscular preadipocytes.

R9: Thank you for your valuable comment. We apologize for the confusion caused by the phrasing. To clarify, our intention was to emphasize that our findings are consistent with previous research in terms of the general effect of LIPG on lipid deposition and cell proliferation, rather than suggesting the use of identical experimental materials or results.
